# Impact of the Domestic Violence Housing First Model on Survivors’ Safety and Housing Stability: 12-Month Findings

**DOI:** 10.1177/08862605221119520

**Published:** 2022-09-02

**Authors:** Cris M. Sullivan, Mayra Guerrero, Cortney Simmons, Gabriela López-Zerón, Oyesola Oluwafunmilayo Ayeni, Adam Farero, Danielle Chiaramonte, Mackenzie Sprecher

**Affiliations:** 1 Michigan State University, East Lansing, MI, USA; 2 Yale University, New Haven, CT, USA; 3 University of Michigan, Ann Arbor, MI, USA

**Keywords:** intimate partner violence, housing, homelessness, intervention, evaluation

## Abstract

Intimate partner violence (IPV) is a widespread and devastating phenomenon resulting in a myriad of long-term consequences for survivors and their children. IPV victimization not only has negative health and economic consequences, it has also been linked to homelessness and housing instability. In response, the Domestic Violence Housing First (DVHF) model is being used in some domestic violence (DV) agencies to help survivors attain safe and stable housing. The model includes using individualized advocacy and/or flexible funding to help survivors meet these goals. Using a longitudinal, quasi-experimental design, the current study involved conducting interviews with survivors and examining agency records to investigate the effectiveness of this model. We hypothesized that survivors who received DVHF would experience less re-abuse and greater housing stability over 12 months compared to those who received services as usual (SAU). The sample included 345 IPV survivors who had been homeless or unstably housed when they approached one of five DV programs for help. Interviews were spaced 6 months apart (when survivors first sought services as well as 6 months and 12 months later). Longitudinal analyses showed that survivors who received the DVHF model reported greater improvements in housing stability at both the 6-month and 12-month time points compared to those receiving SAU. At the 12-month time point, survivors who had received DVHF reported decreased physical, psychological, and economic abuse, as well as the use of their children against them as a form of abuse. This study adds to a growing body of evidence supporting this model’s effectiveness and adds to our understanding of factors impacting the long-term housing stability and safety for IPV survivors.

## Introduction

Intimate partner violence (IPV) is both common and devastating worldwide (Bott et al., 2019; Breiding, 2014; Smith et al., 2018), with women being more severely abused than men and suffering more dire consequences (Breiding, 2014). Types of IPV that can occur while in an intimate relationship with the person causing harm or post-separation can include physical abuse, emotional abuse, sexual abuse, stalking, and economic abuse (Adams et al., 2020; Krebs et al., 2011). Survivors often experience multiple types of victimization within a single intimate relationship (Krebs et al., 2011). It has been well-established that IPV victimization leads to negative mental health consequences for many survivors (Ahmadabadi et al., 2020; Beydoun et al., 2012; Chandan et al., 2020; Trevillion et al., 2012). Survivors are also at increased risk of long-term injuries and other physical health problems (Pritchard et al., 2017; Valera, 2018). Only in recent years has IPV victimization been established as a major contributor to housing instability and homelessness (Broll & Huey, 2020; Chan et al., 2021; Dillon et al., 2016; Pavao et al., 2007). In response, domestic violence (DV) victim service agencies have amplified their efforts in helping survivors attain safe and stable housing. The current study explored how one such effort impacted IPV survivors’ safety and housing stability over the course of 1 year.

### IPV and Housing Instability

IPV may be a direct precursor of housing instability when a survivor is forced to leave their home without the financial means to support themselves or when the abusive partner stops financially contributing to the household. Survivors are too often faced with the choice of either being housed yet unsafe, or safe but unhoused (O’Campo et al., 2016). Other pathways through which IPV can lead to housing instability or homelessness include, for example, when the abusive partner or ex-partner prevents the survivor from paying their bills, ruins the survivor’s credit, causes severe enough physical or emotional injuries that maintaining a household is not possible, or continually stalks and threatens the survivor so that they keep losing residences (Adams et al., 2013; 2020; Lacey et al., 2013). Coupled with the steep decline in affordable housing in the United States (Shaw, 2020), as well as structural inequities that further burden minoritized individuals and communities (Olivet et al., 2021; Velonis et al., 2017; Waller et al., 2022), these factors put IPV survivors at significant risk of housing instability and homelessness.

### Efforts to Help IPV Survivors Attain Safe and Stable Housing

Increasingly, DV service agencies have intensified their efforts to help survivors not just with safety, recovery, and obtaining justice, but with accessing safe housing. This may involve helping them stay safely in their own homes, or it may require finding new housing (Sullivan, Bomsta, et al., 2019; Sullivan, López-Zerón, et al., 2019). One intervention model that more-and-more DV agencies are adopting is Domestic Violence Housing First (DVHF; Sullivan & Olsen, 2016). DVHF is an adaptation of the Housing First (HF) model, which was first designed to help homeless individuals obtain permanent housing (Tsemberis, 2010). Since HF was originally developed for single adults struggling with mental health disorders and addictions, it needed to be adapted for IPV survivors (Sullivan & Olsen, 2016). Adaptations include focusing on safety and trauma and emphasizing social and emotional well-being over harm reduction (Klein et al., 2019; Sullivan, 2018; Sullivan & Olsen, 2016). The DVHF model also recognizes that some survivors may want time in a secure, supportive environment such as a DV shelter or transitional housing before living in their own housing (Wood et al., 2022). An essential element of the DVHF model is that advocates work creatively and proactively in the community, helping survivors obtain safe housing at their own pace. Advocates also support IPV survivors with other issues impacting their safety and well-being (e.g., employment, education).

The other component of DVHF involves providing survivors with cash assistance (Sullivan & Olsen, 2016). Survivors may need financial help directly related to housing (e.g., paying the security deposit, clearing up rent arrears). They may also need financial help with other issues that can enhance their safety and well-being as well as housing stability—such as obtaining legal documents or repairing their vehicles to avoid losing their jobs. Having flexible cash assistance for survivors is consistent with the philosophy of DV agencies to offer survivor-driven services (Cattaneo et al., 2020; Davies & Lyon, 2014), yet is not always available.

The DVHF model was predicated on prior evidence demonstrating that advocacy and cash assistance positively impact survivors’ well-being. Survivor-centered advocacy has been shown to increase survivors’ social support, quality of life, and access to community resources, while decreasing depression and re-abuse (Bybee & Sullivan, 2002). Providing cash assistance to survivors has also been found to lead to increased safety and housing stability (Sullivan, Bomsta, et al., 2019).

### The Current Study

This research is part of a longitudinal study examining the impact of DVHF on IPV survivors over 2 years. Earlier analyses established that those receiving DVHF reported greater housing stability and lower economic abuse than did those receiving services as usual (SAU) 6 months after contacting a DV agency (Sullivan et al., 2022). The current study builds upon this initial evidence by examining the extent to which DVHF contributes to survivors’ safety and housing stability across 1 year. We hypothesized that survivors receiving the DVHF model would show greater improvement at 12 months on each of these dimensions compared to survivors receiving SAU.

## Method

The current research includes baseline, 6-month, and 12-month data from an ongoing longitudinal study examining the extent to which the DVHF model improves the lives of unstably housed IPV survivors over the course of 2 years.

### Participants and Procedures

Participants were recruited from five DV agencies (three rural and two urban) in a Pacific Northwest state in the United States. During the recruitment period, DV agency staff informed all new clients who were either homeless or unstably housed about the study. The final sample included 406 survivors (93% of those eligible). We conducted interviews in English (88%) or Spanish (12%), depending on the survivor’s preference. Interviews were conducted in-person or over the phone, again based on survivors’ preference. Participants were paid $50 for each interview, and Institutional Review Board approval was granted through the first author’s university.

### Study Design

We purposefully chose to use a quasi-experimental study design conducted within the community, in order to maximize ecological validity. Instead of randomizing survivors into DVHF and SAU groups, all survivors entering services were eligible to participate in the study. This design assured that we would capture the typical variability in service delivery (e.g., some survivors would enter services when DV agencies had capacity to provide DVHF and others would receive SAU). Those who received SAU (e.g., support groups, counseling, safety planning, shelter, or other forms of advocacy not related to housing stability) were compared to those who received the DVHF model (housing-focused advocacy and/or cash assistance, regardless of other services they may have also received). For more information about the study design, refer to the study by Sullivan et al. (2021).

### Measures

In addition to capturing typical sociodemographic data, interviews included the following measures of services received, safety, and housing stability:

#### Services received

Participants were asked about the services they had received from the agency and were specifically asked (yes/no) if a staff member had helped them “work on housing and getting other things” they needed from the community.

#### Cash assistance received

Each agency recorded when they provided cash assistance, how much they provided, and how it was used.

#### Physical abuse, emotional abuse, stalking, and sexual abuse

These forms of IPV were assessed using the Composite Abuse Scale (CAS; Loxton et al., 2013). The original CAS response options ranged from *daily* to *never.* These were modified to be compatible with interviews occurring every 6 months: 0 = *never*, 1 = *once*, 2 = *several times or between 2-3x in the last 6 months*, 3 = *once a month*, 4 = *once a week*, and 5 = *daily*. Two CAS items (*hang around outside your house* and *harass you at work*) were replaced with a new item that was more inclusive of stalking by an intimate partner (*repeatedly follow you, phone you, and/or show up at your house/work/other place*). Four items were added to measure types of abuse not adequately measured in the original scale: (1) *stalk you*, (2) *strangle you*, (3) *demand sex whether you wanted to or not*, and (4) *force sexual activity*. Subscales were computed by averaging their respective items. Cronbach’s α was .95 for the entire scale; subscales ranged from .84 to .92.

#### Economic abuse

Economic abuse was measured with the 14-item Revised Scale of Economic Abuse (Adams et al., 2020). Items included asking how often in the past 6 months the abusive partner/ex-partner would “make you take out a loan to buy something on credit when you didn’t want to,” and “make you ask them for money.” Response options ranged from 0 = *never* to 4 =*quite often*. Cronbach’s α was .91.

#### Abuser’s use of children as a tactic of IPV

The frequency with which participants’ abusers had used the participants’ children against them as a form of manipulation or control was assessed using the 7-item Use of Children to Control scale (Beeble et al., 2007). Only parents of minor children were asked these questions (*n* = 297). The scale consisted of items measuring how often in the previous 6 months the abuser had used the children to stay in their lives, harass, intimidate, track, or frighten them, as well as tried to turn the kids against them or convince them to take the abuser back. Participants reported frequency on a 5-point Likert-type scale from 0 = *never* to 4 = *quite often*. Cronbach’s α for the scale was .87.

#### Housing instability

A 7-item Housing Instability Scale (HIS) was created that included 6 items from the 10-item Housing Instability Index (HII; Rollins et al., 2012) as well as: “In the last 6 months, have you been homeless or had to live with family or friends to avoid being homeless?” Four items from the HII were removed because they pertained to landlords or renting, and many of this study’s participants did not have landlords. The final scale included five originally dichotomous *yes/no* responses and 2 items that were recoded to be dichotomous. Scores range from 0 to 7, with higher scores denoting greater housing instability. The HIS has established both concurrent and predictive validity, as well as scalar equivalence over time, across both the English and Spanish versions (Farero et al., under review). Cronbach’s α for the HIS at baseline, 6-month and 12-month follow up all suggest adequate internal consistency (.66, .71, and .77 respectively). Cronbach’s α for the sum score averaged over each time point was .79.

### Analyses

Retention was 92% at 6-month follow-up and 91% at 12-month follow-up. Survivors retained in the study were comparable to those not retained with regard to race, ethnicity, age, history of abuse, history of homelessness, relationship status, number of children, and mental health symptomatology.

#### Determining who received DVHF versus SAU

Participant interviews and agency data were used to determine, at the 6-month time point, who had received the DVHF model and who had received SAU. Thirty participants were removed from analyses because they had received no services after their initial intake at the DV agency. Thirty-six percent of the remaining participants were considered to have received SAU because they had received services but (1) had not received housing-focused advocacy, despite needing such help; and (2) had not received cash assistance. Survivors who had received housing-focused advocacy and/or cash assistance were categorized as having received DVHF (64%) (see Sullivan et al., 2022 for more detail). Eight participants who were not interviewed at the 6-month follow-up were regained into the study at the 12-month follow-up. Based on their agency records, we determined that, between baseline and the 6-month interview, two had received no services, three had received SAU, and three had received the DVHF model.

#### Longitudinal analyses

All study participants who had received services and who were retained in the study at 6 and/or 12 months were included in the longitudinal analyses. To control for baseline group differences that could affect outcomes, we calculated inverse probability weighted (IPW) estimators for each participant (Hernán & Robins, 2020; Joffee et al., 2004). IPW estimators enable investigators to account for selection bias by estimating two models simultaneously: an intervention model that includes variables influencing group membership and an outcome model that includes the intervention and other relevant covariates as predictors. Six additional participants were dropped from analyses due to missing data on one or more covariates used to generate the inverse probability weights. This resulted in an analytic sample of 345 at 12 months.

A stepwise selection procedure was used to identify covariates to include in the models (Gareth et al., 2013). This process was conducted for each outcome at 12 months, allowing for parsimonious outcome models to be tested across the three time points. When comparing outcome models with and without covariates, no changes in the relationships between study variables were found. Models with covariates are reported.

**Figure 1.**
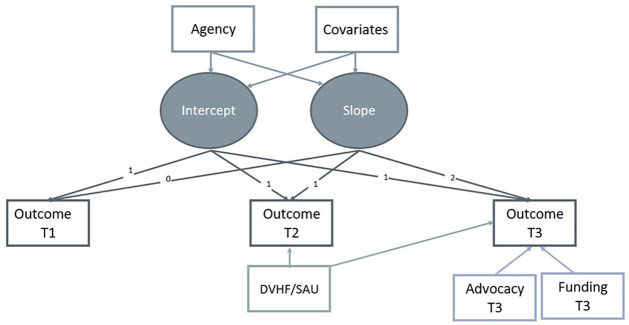
Latent growth curve model.

**Figure 2.**
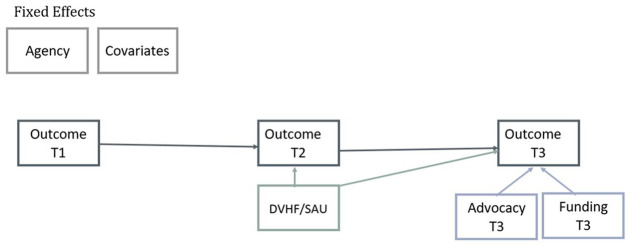
Path model.

Latent growth curve and path analyses were used to examine outcomes 6 and 12 months after survivors sought services, comparing those who had received DVHF during the first 6 months of the study to those who had received SAU. Latent growth curve analyses were used to compare changes in housing stability. An unconditional growth model was tested first to determine the variability in initial levels of housing stability and average change in housing stability over time for the overall sample. Since analyses were confined to three time points, a linear trajectory in growth was selected for evaluating change in housing stability. A conditional growth model was tested second to determine whether housing stability differed among those who received DVHF and those who received SAU (Figure 1). To account for the steep decline in abuse that occurred between baseline and 6 months, path model analyses were conducted for all abuse outcomes (Figure 2). Across all models, agency and advocate were treated as fixed effects, and IPW estimators were included as sampling weights. Covariates that were chosen through the data-based selection procedure were included in the models as time-invariant predictors. Additionally, some participants continued to receive services between 6 and 12 months after seeking help, so models included whether participants received funding and advocacy during this timeframe as covariates. Models also controlled for each participant’s baseline levels of the outcome under investigation. Overall fit was assessed using the χ^2^ likelihood ratio statistic. Given that the χ^2^ statistic is sensitive to sample size and that it is not uncommon for well-fitting models to have significant values when the number of observations exceeds the degrees of freedom (Hu & Bentler, 1995), we also used the comparative fit index (CFI) and the root mean square residual (RMSEA) as additional indicators of model fit (recommended thresholds: CFI ≥ .90; RMSEA < .08; Hu & Bentler, 1999). All longitudinal analyses were conducted in R 4.1. (R Core Team, 2021) using the lavaan package (Rosseel, 2012). Missing data were handled through full information maximum-likelihood estimation.

## Results

The analytic sample was largely female (97%) and heterosexual (87%). Average age was 34.5 years old, with ages ranging from 19 to 62. Sixty-four percent reported a minoritized racial/ethnic identity: Hispanic/Latinx (35%), Black (18%), U.S. Indigenous (11%), Asian (5%), and/or Middle Eastern (1%). Sixteen percent of the minority survivors reported multiracial or multi-ethnoracial identities. The remaining 36% were non-Hispanic White. Table 1 presents detailed participant sociodemographics.

**Table 1.** Sociodemographics of Participants in Longitudinal Analyses (*N* = 345).

	DVHF (*n* = 220)	SAU (*n* = 125)	Total (*N* = 345)
Age (*mean; SD*)	34.49 (9.09)	34.63 (8.99)	34.54 (9.05)
	% (*n*)	% (*n*)	% (*n*)
Female	98.2 (216)	95.2 (119)	97.1 (335)
Heterosexual	88.6 (195)	83.9 (104)	86.9 (299)
Race/ethnicity (could choose all that apply)			
Non-Hispanic White only	31.8 (70)	42.4 (53)	35.7 (123)
Hispanic/Latinx	33.6 (74)	38.4 (48)	35.4 (122)
Black	23.6 (52)	8.8 (11)	18.3 (63)
U.S. Indigenous	10.0 (22)	12.0 (15)	10.7 (37)
Asian	5.5 (12)	3.2 (4)	4.6 (16)
Middle Eastern	1.8 (4)	0	1.2 (4)
Multiracial/multiethnic	14.1 (31)	19.2 (24)	15.9 (55)
U.S. Citizen	79.1 (174)	87.2 (109)	82.0 (283)
Primary Language English	80.0 (176)	81.6 (102)	80.6 (278)
Parenting minor children	78.2 (172)	68.0 (85)	74.5 (257)
Employed in the last 6 months	60.9 (134)	55.2 (69)	58.8 (203)
Education			
Less than high school	24.1 (53)	34.4 (43)	27.8 (96)
High school graduate/GED	22.2 (49)	22.4 (28)	22.3 (77)
Vocational/training certificate	9.5 (21)	7.2 (9)	8.7 (30)
Some college	22.7 (50)	16.0 (20)	20.3 (70)
Associate degree	7.3 (16)	8.0 (10)	7.5 (26)
Bachelor’s degree	9.5 (21)	6.4 (8)	8.4 (29)
Advanced degree	4.5 (10)	5.6 (7)	4.9 (17)
Prior history of homelessness	71.8 (158)	77.6 (97)	73.9 (255)
Homeless as a child/adolescent	31.6 (50)	28.0 (35)	33.3 (85)

*Note*. DVHF = Domestic Violence Housing First; GED = General Education Diploma; SAU = services as usual.

### Services Received in the First 6 Months After Seeking Services

Thirty-six percent of the analytic sample (*n* = 125) received SAU from the agency between baseline and 6 months (e.g., counseling, support groups, referrals, shelter). Within the DVHF sample (*n* = 220; 64%), 29% received housing-focused advocacy but no funding, 17% received funding but no housing-focused advocacy, and 54% received both housing-focused advocacy and funding. These participants may or may not have also received other agency services. After accounting for advocate and agency, logistic regressions confirmed that minority status was not a significant predictor of services received (Sullivan et al., 2022).

### Examining Continued Use of Services 12 Months After Seeking Services

DV agencies do not provide time-limited services (with the exception of some having time limits on shelter or transitional housing stays). As such, some survivors receive short-term services while others receive longer term or intermittent services. Given this natural variability, we examined how many study participants received services between the 6-month and 12-month follow-up time frame. Not surprisingly given the nature of DV services, only about two in five of the study participants (42%) received services from the recruiting agency during this time. Of participants who received SAU during the first 6 months, 30% received services between 6-month and 12-month follow-up (see Table 2). Just under half (49%) of participants who received the DVHF intervention during the first 6 months of this study continued to receive services between the 6-month and 12-month follow-up. Of participants who continued to receive services, those who had received DVHF were more likely than those who had received SAU to receive advocacy services in the 6-to-12-month timeframe (83% vs. 71%). A much higher percentage of those who had received DVHF in the first 6 months after seeking services received direct financial assistance when compared to those who had received SAU (41% vs. 14%).

**Table 2.** Services Received from 6 to 12 Months (*n* = 334*).

	Services Between 6 and 12 Months				
Initial Grouping at 6 Months	No Services	Services, No Advocacy or Funds	Advocacy, No Funds	Funds, No Advocacy	Advocacy and Funds
SAU (36%; *n* = 120)	70% *n* = 84	8% *n* = 9	18% *n* = 22	<1% *n* = 1	3% *n* = 4
DVHF (64%; *n* = 214)	51% *n* = 109	5% *n* = 11	24% *n* = 51	3% *n* = 7	17% *n* = 36
		Of Those Receiving Services:			
SAU (*n* = 36)		25% *n* = 9	61% *n* = 22	3% *n* = 1	11% *n* = 4
DVHF (*n* = 105)		10% *n* = 11	49% *n* = 51	7% *n* = 7	34% *n* = 36

*Note*. DVHF = Domestic Violence Housing First; SAU = services as usual.

* Five participants who had received SAU and six participants who had received DVHF did not complete 12-month interviews and are not included in this table.

### DVHF Impact on Housing Stability

The unconditional latent growth model predicting housing stability demonstrated good model fit on indices not dependent on sample size, χ^2^(1) = 0.004, *p* < .951; CFI = 1.00; RMSEA = 0.00. The intercept estimate was 4.75 and differed significantly from zero (*p* < .001), suggesting that the average levels of housing stability at baseline differed across individuals. The slope estimate was negative (−1.09), and significant (*p* < .001), suggesting that the average rate of change on housing stability for the overall sample differed across time, with participants becoming more stably housed overall. The significant slope variance estimate (0.57, *p* < .005) indicates that there are between-individual differences in patterns of growth over time on housing stability. A nonsignificant intercept and slope covariance suggests that while individuals differed on initial values of housing stability, changes in housing stability over time were not associated with participants’ initial starting levels on this outcome. The conditional growth model demonstrated good model fit on indices not dependent on sample size (see Table 4). DVHF recipients reported greater housing stability compared to those who had received SAU at both 6 months (*b* = −0.82, β = −0.21, *SE* = 0.17, *p* < .001) and 12 months (*b* = −1.03, β = −0.25, *SE* = 0.19, *p* < .001), with small effect sizes.

### DVHF Impact on Safety

Unconditional models were tested to evaluate the stability of safety outcomes over time: total abuse between baseline and 6 months (*b* = 0.24, β = 0.35, *SE* = 0.04, *p* < .001) and 6 to 12 months (*b* = 0.45, β = 0.53, *SE* = 0.09, *p* < .001); physical abuse between baseline and 6 months (*b* = 0.18, β = 0.32, *SE* = 0.04, *p* < .001) and 6 to 12 months (*b* = 0.23, β = 0.29, *SE* = 0.09, *p* < .001); sexual abuse between baseline and 6 months (*b* = 0.10, β = 0.22, *SE* = 0.03, *p* < .001) and 6 to 12 months (*b* = 0.36, β = 0.46, *SE* = 0.13, *p* < .001); emotional abuse between baseline and 6 months (*b* = 0.24, β = 0.33, *SE* = 0.04, *p* < .001) and 6 to 12 months (*b* = 0.43, β = 0.51, *SE* = 0.09, *p* < .001); economic abuse between baseline and 6 months (*b* = 0.26, β = 0.31, *SE* = 0.04, *p* < .001) and 6 to 12 months (*b* = 0.58, β = 0.69, *SE* = 0.06, *p* < .001); stalking between baseline and 6 months (*b* = 0.32, β = 0.36, *SE* = 0.08, *p* < .001) and 6 to 12 months (*b* = 0.55, β = 0.64, *SE* = 0.07, *p* < .001); and use of child as a abuse tactic between baseline and 6 months (*b* = 0.60, β = 0.56, *SE* = 0.06, *p* < .001) and 6 to 12 months (*b* = 0.75, β = 0.60, *SE* = 0.06, *p* < .001) were positively and significantly related. Experience of abuse declined across the entire sample over time (see Table 3), with particularly sharp declines in IPV occurring from baseline to 6 months. Path models evaluating the impact of DVHF on re-abuse demonstrated good to acceptable model fit (Table 4). There were no group differences on abuse outcomes at 6 months. Four significant differences emerged at 12 months: physical abuse, emotional abuse, economic abuse, and use of the children as an abuse tactic. Those who received DVHF reported significant decreases in physical abuse (*b* = -0.14, β = −0.14, *SE* = 0.06, *p* < .05), economic abuse (*b* = −0.13, β = −0.09, *SE* = 0.06, *p* < .05), emotional abuse (*b* = −0.21, β = −0.13, *SE* = 0.10, *p* < .05), and use of child as an abuse tactic (*b* = -0.19, β = −0.08, *SE* = 0.09, *p* < .05) compared to those receiving SAU. All effect sizes were small (see Table 4).

**Table 3.** Descriptives of Housing Instability and IPV Outcomes Over Time.

Outcomes	DVHF					
	Baseline		6 Months		12 Months	
	*M*	*SD*	*M*	*SD*	*M*	*SD*
DVHF						
Housing instability	4.48	1.66	2.88	1.98	2.02	1.91
Total abuse	1.58	1.09	0.47	0.70	0.33	0.54
Physical abuse	1.22	1.03	0.26	0.60	0.13	0.40
Sexual abuse	1.09	1.47	0.16	0.61	0.09	0.42
Emotional abuse	1.97	1.32	0.51	0.85	0.41	0.68
Stalking/harassment	2.05	1.54	0.94	1.21	0.71	1.07
Economic abuse	1.4	1.05	0.38	0.71	0.24	0.57
Use of children	1.75	1.12	1.11	1.22	0.98	1.27
SAU						
Housing instability	5.28	1.53	4.26	1.80	3.22	2.23
Total abuse	1.89	1.17	0.68	0.77	0.55	0.74
Physical abuse	1.43	1.18	0.35	0.63	0.27	0.67
Sexual abuse	1.3	1.56	0.24	0.77	0.21	0.71
Emotional abuse	2.29	1.25	0.74	1.00	0.68	0.95
Stalking/harassment	2.53	1.69	1.38	1.48	1.10	1.32
Economic abuse	1.59	1.03	0.67	1.02	0.48	0.81
Use of children	1.62	1.11	1.20	1.23	1.19	1.22
Total						
Housing instability	4.77	1.66	3.37	2.03	2.45	2.11
Total abuse	1.69	1.13	0.54	0.73	0.41	0.63
Physical abuse	1.29	1.08	0.29	0.61	0.18	0.52
Sexual abuse	1.16	1.50	0.19	0.67	0.13	0.54
Emotional abuse	2.08	1.30	0.59	0.91	0.51	0.78
Stalking/harrassment	2.22	1.61	1.10	1.33	0.85	1.18
Economic abuse	1.47	1.04	0.48	0.84	0.32	0.68
Use of children	1.71	1.12	1.14	1.22	1.06	1.25

*Note*. IPV = intimate partner violence; DVHF = Domestic Violence Housing First; SAU = services as usual.

**Table 4.** Structural Equation Model Results Comparing DVHF and SAU at 12 Months.

Outcomes	*b*	β	*SE*	*p*-Value	95% CI		Model Fit Indices			
					Lower Bound	Upper Bound	χ^2^	*p*-Value	CFI	RMSEA
Housing instability										
6 months*	−0.818	−0.205	0.168	<.001	−1.147	−0.489	34.235	.008	0.996	.066
12 months*	−1.026	−0.248	0.194	<.001	−1.470	−0.489				
Economic abuse										
6 months	−0.077	−0.044	0.135	.567	−0.324	0.187	7.679	.263	1.000	.030
12 months*	−0.129	−0.088	0.063	.040	−0.252	−0.006				
Composite abuse scale total										
6 months	−0.039	−0.026	0.078	.621	−0.192	0.115	1.297	.972	1.000	.000
12 months	−0.160	−0.127	0.070	.022	−0.297	−0.023				
Physical abuse							7.455	.281	0.988	.026
6 months	0.047	0.039	0.064	.457	−0.077	0.172				
12 months*	−0.141	−0.144	0.060	.019	−0.259	–0.023				
Emotional abuse							1.367	.968	1.000	.000
6 months	−0.015	−0.008	0.095	.872	−0.201	0.171				
12 months*	−0.214	−0.132	0.095	.025	−0.400	−0.027				
Sexual abuse							7.417	.284	1.000	.026
6 months	−0.003	−0.002	0.073	.967	−0.147	0.141				
12 months	−0.061	−0.058	0.047	.192	−0.153	0.031				
Stalking							2.703	.845	1.000	.000
6 months	−0.160	−0.056	0.196	.415	−0.544	0.224				
12 months	−0.206	−0.085	0.105	.051	−0.412	0.001				
Use of children							10.864	.093	0.982	.064
6 months	−0.077	−0.031	0.132	.562	−0.335	0.182				
12 months*	−0.186	−0.076	0.093	.045	−0.368	−0.004				

*Note*. SAU is the reference group. Unstandardized coefficients (*b*), standardized coefficients (β, robust standard errors (*SE*), and 95% confidence intervals (CI) are reported. DVHF = Domestic Violence Housing First; SAU = services as usual; CFI = comparative fit index; RMSEA = root mean square residual

* *p* < .05.

## Discussion

This study provides evidence that the DVHF model is more effective than SAU in enhancing IPV survivors’ housing stability over time. Those who received DVHF reported higher housing stability at both 6-month and 12-month follow-up compared to those receiving SAU. This finding corroborates a prior study that noted increased housing stability among IPV survivors who received direct financial help (Sullivan, Bomsta, et al., 2019), as well as a pilot study of the DVHF model (Mbilinyi, 2015). It is promising to see that this model increased housing stability relatively quickly (within 6 months of approaching an agency for help) and that housing stability was maintained 1 year later. This suggests that, while DV agency “services as usual” are likely helpful in improving survivors’ safety and socio-emotional well-being (Sullivan, 2018; Sullivan & Virden, 2017), DVHF’s specific emphasis on housing-focused advocacy and funding may be key in helping survivors achieve housing stability.

The second, but equally important, goal of the DVHF model is to enhance survivor safety so that they do not have to choose between being safe or being housed. While there were no significant group differences on safety across the first 6 months of the study, there was a sharp decline in abuse across the full sample. This is likely due to having received immediate support, safety planning and protection from the DV agencies, although in the absence of study participants who sought no help at all, we can only deduce this. This finding does, however, corroborate prior evidence that DV agencies make a positive difference in the lives of IPV survivors (Davies & Lyon, 2014; Sabri et al., 2022; Sullivan & Virden, 2017; Wood et al., 2022).

Interestingly, significant group differences emerged for four types of abuse at the 12-month follow-up. Those who had received DVHF reported less physical abuse, emotional abuse, economic abuse, and use of the children as an abuse tactic compared to those who had received SAU. Understanding this delayed effect of DVHF will require further analyses and may become clearer when examining additional time points. Prior research has linked homelessness to increased risk of abuse (e.g., Calvo et al., 2021; Gilroy et al., 2016), so it may be that housing stability is a precursor to safety. The difference may also relate to the fact that more of the participants who had received DVHF continued to be receiving support between the 6 and 12-month time points. A tenet of the DVHF model is to provide services for as long as they are needed (Sullivan & Olsen, 2016). In the current study, more of the survivors who had received DVHF continued to receive services compared to those who had received SAU (49% vs. 30%), suggesting that this component of the model was being followed. When examining services received by survivors between 6 and 12 months after approaching an agency for help, those who had received the DVHF model were also more likely to receive advocacy and funding as components of their services compared to those who had received SAU.

### Study Limitations

Study results should be considered in light of limitations. Both practical and ethical considerations led us to choose a quasi-experimental design over a randomized control trial (for additional detail, see Sullivan et al., 2021), and it is important to note that survivors were not randomized into condition. While we took judicious steps to ensure the accuracy of service classification, and controlled for preexisting group differences, unidentified relationships may have contributed to service receipt or outcomes achieved. For example, some survivors may have been offered housing-focused advocacy but declined this service because they either had not decided what they wanted to do next, or they already had housing lined up through other means. The extent to which participants were receiving help from other agencies, families, and friends is not known and could have influenced what services they wanted as well as their ability to achieve safe and stable housing.

All the study participants were unstably housed or homeless at the start of the study, and all had sought help from a DV program. Further, while the study was racially and ethnically diverse, few participants were Indigenous or of Asian or Arab descent. Most participants also identified as heterosexual, cisgender women. Generalizability of these findings should be considered within these limitations.

The current study was only able to test whether the DVHF model as a whole improved housing and safety outcomes. The model’s two components—housing-focused advocacy and flexible funding—cannot be analyzed separately due to the nature of the DVHF model itself. The model is predicated on being survivor-driven, with services and/or funding being provided as requested and needed by different survivors. Some survivors only want brief services while others may require help over months or years. Some need more financial help than others. In some cases, the DV program can locate other sources of funding or help for the survivor rather than using the DV agency’s resources. Future studies may help tease out the relative importance of advocacy versus funding, but these need to always be weighed in light of survivors’ needs.

It is also imperative to understand more about SAU. DV agencies offer a wide range of services, from brief referrals to long-term counseling and support groups. Here, too, services are supposed to match the self-identified needs of survivors, which makes examining service impact especially complex (Sullivan, 2011). Within this sample of survivors who had received DV services, both groups showed a sharp decrease in abuse over the first 6 months of the study. This speaks to the importance of DV services overall and suggests that different services may be related to different outcomes over time.

### Implications

Findings from this study have both research and policy implications. This is the first longitudinal study to examine the impact of the DVHF model over time, and clearly much more research is needed. Studies with even more diverse samples, across different geographic regions, and that employ a variety of methodologies, will help create a more comprehensive understanding of how this model works, for whom, and under what conditions.

The policy implications of this study are considerable. If this model contributes to survivors’ safety and housing stability not just immediately but over time, it deserves more financial investment from funders so that it can be more broadly implemented. It is time-consuming for advocates to work closely with survivors in the community locating and obtaining safe housing. Further, such advocacy efforts generally involve focusing on interrelated issues such as employment, transportation, and health care, requiring advocates to have a variety of skills and knowledge to be effective (Sullivan & Goodman, 2019). It is unfortunately extremely common for DV agencies to be under-staffed and to experience high staff turnover, which can limit the amount of time advocates can work with survivors. A stronger investment in DV agencies, so they can hire, train, and supervise more advocates, could greatly reduce survivors’ risk of re-abuse and enhance their housing stability. Further, a stronger investment toward strengthening service integration between DV and homeless systems is necessary to ensure survivors’ housing needs are better addressed by a more seamless, responsive, and coordinated system of care.

In addition to investing in more personnel to provide housing-focused advocacy, public and private funders should consider supporting DV agencies’ use of flexible financial assistance for their clientele. Every IPV survivor who seeks help has their own unique set of circumstances and needs, which is why a major tenet of DV programs is to provide survivor-driven services (Davies & Lyon, 2014; Goodman & Espstein, 2008; Sullivan, 2018). It is difficult to individualize services to IPV survivors without funding that can be used flexibly to address their needs. Some survivors need their rent paid for a period of time while they can stabilize their lives, or they may lack a security deposit to secure new housing. Others may need help paying for car repairs in order to keep their jobs and get their children to school. Not all survivors need thousands or even hundreds of dollars, and even small amounts of funding can sometimes be life-altering. We have heard of numerous instances where a survivor needed less than a hundred dollars for a work uniform, an occupational license, or to cover a similar expense that, while not expensive, was critical to their employment, education, health, or housing.

Governmental sources of funding in the United States are generally guided by whether expenditures are cost effective or “a good use of taxpayer dollars” (Parsell et al., 2017). Given the high cost-burden on society when individuals experience homelessness and housing instability (Culhane, 2008; Wright et al., 2016), investing in a model that increases safe and stable housing seems particularly justifiable. Agency leadership and policymakers must work together to ensure services effectively address survivors’ unique needs. This work can often be complex and time-consuming (Sullivan et al., 2019) but, as this study indicates, survivor-driven, housing-focused advocacy, and flexible cash assistance are critical components of the support survivors need to obtain and maintain safe and stable housing.
